# The Immune Resistance Signature of Acute Myeloid Leukemia and Current Immunotherapy Strategies

**DOI:** 10.3390/cancers16152615

**Published:** 2024-07-23

**Authors:** Daniel J. Chandra, Bernhard Alber, Jennifer N. Saultz

**Affiliations:** 1 Division of Hematology/Medical Oncology, Oregon Health & Science University, Portland, OR 97239, USA; chandrda@ohsu.edu; 2 Knight Cancer Institute, Oregon Health & Science University, Portland, OR 97239, USA; alber@ohsu.edu

**Keywords:** AML, immunotherapy, natural killer cell, T cell

## Abstract

**Simple Summary:**

Acute myeloid leukemia (AML) is an aggressive cancer of stem cells originating in the bone marrow. Increasing evidence suggests that AML survival and relapse are controlled by genetic alterations, as well as a functional interaction between the tumor and the immune system. In this review, we will discuss the biology of immune evasion in AML and explore the current landscape of immune-based therapies. We argue that a better understanding of the immune microenvironment in AML could help predict responses to immunotherapy and guide decisions for future treatment.

**Abstract:**

Acute myeloid leukemia (AML) is a complex hematopoietic clonal disorder with limited curative options beyond stem cell transplantation. The success of transplant is intimately linked with the graft versus leukemia effect from the alloreactive donor immune cells including, T and NK cells. The immune system plays a dynamic role in leukemia survival and resistance. Despite our growing understanding of the immune microenvironment, responses to immune-based therapies differ greatly between patients. Herein, we review the biology of immune evasion mechanisms in AML, discuss the current landscape of immunotherapeutic strategies, and discuss the implications of therapeutic targets. This review focuses on T and NK cell-based therapy, including modified and non-modified NK cells, CAR-T and CAR-NK cells, antibodies, and checkpoint blockades. Understanding the complex interchange between immune tolerance and the emergence of tumor resistance will improve patient outcomes.

## 1. Introduction

Acute myeloid leukemia (AML) is an aggressive disease characterized by the proliferation of blasts in the bone marrow and clonal expansion [1]. Recurrent genetic mutations lead to leukemia initiation; however, impaired immune surveillance mechanisms ultimately allow for growth and disease [1]. A plethora of novel immune-based treatments have emerged in the last 20 years for AML, including both targeted and non-targeted approaches [2]. Many of these treatments offer short-term responses, and none to date have been curative. Allogeneic hematopoietic cell transplant (alloHSCT) is the oldest form of immune therapy, relying on the graft vs. leukemia effect of the donor immune cells to eradicate and prevent relapse. Unfortunately, relapse after alloHSCT is still common, ranging from 20 to 50% of patients, with 2-year survival rates below 20% independent from the choice of salvage therapy [3]. To address this unmet need, cellular-based immune therapies have emerged, such as chimeric antigen receptor (CAR) T or natural killer (NK) cell therapy, allogeneic non-modified NK cells, NKT cells, antibodies, bispecific/trispecific T-cell engagers (BiTEs/TRiKEs), and checkpoint inhibitors. Immune checkpoints are activating or inhibitory receptor/ligand synapses between an effector cell (T or NK cell) and its ligand and are needed to elicit an immune response (i.e., PD1-PDL1) [4]. Although treatments, including CAR-T, have become mainstream in treating relapsed B cell acute lymphoblastic leukemia [5] and B cell lymphomas [6], they have been less effective in AML due to the lack of a direct antigen target. We are at an important juncture within the field of immunotherapies for AML with enormous potential and promise. A detailed understanding of the immune microenvironment is critical for determining the success or failure of cellular therapy. Herein, we will review what is known regarding mechanisms of immune escape in AML and discuss therapeutic strategies to improve outcomes in patients.

### 1.1. Overview of Mechanisms of Immune Evasion in AML

**Figure 1:**
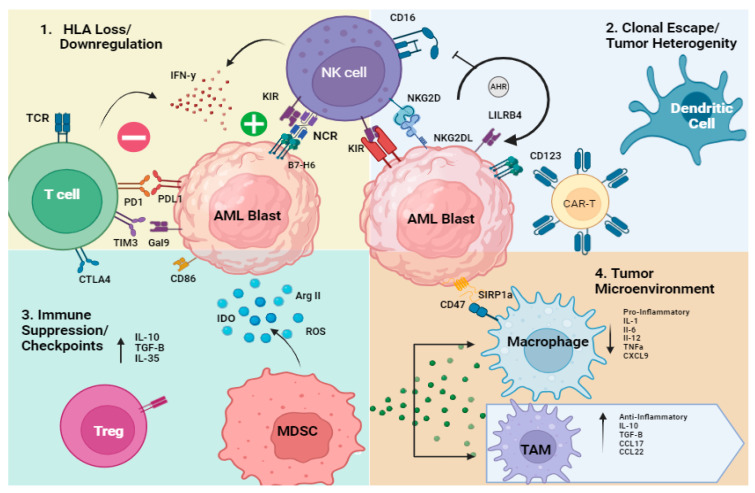
Immune evasion strategies. (1) AML cells downregulate or have a genetic loss of human leukocyte antigen (HLA), which prevents T cell recognition through the TCR but may activate NK cells due to lack of binding the inhibitory KIR and activation through NK cell NCRs. In addition, NK cells and T cells secrete IFN-γ which regulates HLA Class I and II expression. (2) Clonal escape/tumor heterogeneity drives immune dysfunction by loss of antigen target (i.e., CD123) or blast maturation state shifts (i.e., AHR driving monocytic maturation) leading to changes in tumor antigen expression such as LILRB4 on monocytic blasts. (3) Immune suppression/checkpoint upregulation on AML blasts including T cell inhibitory ligands (PD1, TIM3, CTLA4) leading to impaired T and NK cell function. (4) Tumor microenvironment (TME) changes with increased immune suppressive cells such as myeloid-derived suppressor cells (MDSCs), tumor-associated macrophages (TAMs), and Regulatory T-cells (Tregs) which secrete cytokines such as IL-10, TGF-B, IL-35 and soluble factors (ROS, IDO, Arg II) which drive immune resistance. TAMs also secrete anti-inflammatory cytokines such as IL-10, TGF-B, CLCL17 and CCCL22. This figure was created with Biorender.com.

AML is driven by oncogenic mutations impacted by epigenetic changes leading to proliferation and survival. Once cancer develops, the progression and clonal expansion relies on immune evasion strategies for survival. T-cell exhaustion, NK cell dysfunction, and complex microenvironmental changes foster a climate supportive of leukemia progression and treatment resistance [7,8,9,10]. The AML niche plays a critical role by allowing crosstalk between blasts, stroma, and immune cells driving defective immune surveillance [7,11]. In this review, we will focus on four main mechanisms of immune evasion strategies highlighted in the literature: (1) genetic or transcriptional loss of human leukocyte antigen (HLA); (2) clonal escape/tumor heterogeneity/oncogenesis; (3) immune suppression (T and NK cells, MDSCs, dendritic cells)/checkpoint upregulation; and (4) altered cytokine milieu/tumor microenvironment (TME) [12,13,14] (Figure 1). Each of these immune alterations is explained in detail below.

1. Genetic or transcriptional loss of HLA

The downregulation or complete genomic loss of HLA is a common form of immune evasion in AML, suggesting that evading T-cell-mediated immune surveillance is a critical step in cancer progression. Major histocompatibility complex (MHC) antigens are found on the surface of cells that are critical for displaying peptide fragments to cytotoxic T cells for activation and tumor recognition [15]. Class I MHC molecules are found on all nucleated cells, including myeloid blasts, while MHC class II is found primarily on antigen-presenting cells (APCs), such as B lymphocytes, dendritic cells, macrophages, monocytes, and endothelial cells. The main MHC class I antigens are HLA-A, HLA-B, and HLA-C, while the class II antigens are HLA-DR, DP, and DQ [16]. Myeloid cells can both process and present self or tumor antigens, allowing for engagement and activation of CD8 T cells through the TCR. When myeloid blasts become leukemic, they often have defective MHC antigen presentation, preventing normal immune surveillance and recognition by T cells. However, this also activates NK cells through enhanced recognition of the tumor and killing via the lack of engagement with the inhibitory killer immunoglobin receptor (KIR) [17]. This potential for immune engagement based on the presence or absence of MHC-I and MHC-II molecules creates opportunities to therapeutically redirect immune cells toward an activated state.

Several studies have characterized the loss of MHC molecules as a major cell survival mechanism in AML [18,19]. In allogeneic transplants, the donor alloreactive T cells and NK cells are responsible for mediating graft vs. leukemia to prevent relapse. Christopher et al. performed detailed RNA sequencing from paired samples of cryopreserved patient bone marrow on initial diagnosis and at relapse. The authors included 15 post-transplant patients, including HLA-matched siblings, HLA-matched and unmatched unrelated donors, and 20 patients who had a relapse after chemotherapy. Analysis of the RNA sequencing and later confirmed with flow cytometry found that the major driver of relapse was the downregulation of MHC class II (DPA1, HLA-DPB1, HLA-DQB1, and HLA-DRB1). In four of the tested post-transplantation relapse samples with decreased expression of MHC class II, the authors show that these samples did not stimulate third-party CD4+ T cells in vitro, suggesting that blocking the ability of the donor CD8+ and CD4+ T cells to recognize the leukemia target prevents recognition through failed antigen presentation [20]. Importantly, they did not identify any relapse-specific gene fusion, particularly fusions involving the MHC class II regulatory gene class II transactivator (CIITA). Thus, the mechanism for the MHC class II downregulation was not elucidated in this study. The expression of oncogenes, including Myc, has been shown to downregulate HLA, but this was not identified in this study [21]. Toffalori et al. analyzed a similar population of relapsed AML patients after alloHSCT by performing gene expression profiles of AML blasts purified from patients at serial time points and found that class II downregulation was a mechanism of relapse in AML related to defective CIITA. This study also found that relapsed blasts have increased suppressive ligands on the cell surface, which hinder immune recognition through T-cell suppression via PD-L1, B7-H3, CD80, and PVRL2 [22]. Both of the above studies, however, show that interferon-γ or checkpoint blockade may counteract this suppression and restore immune surveillance. In the context of HLA haploidentical transplantation, AML relapse is driven by the loss of the mismatched haplotype. Vago et al. performed a detailed examination of genetic changes in donor–host haplotypes after relapse using polymerase chain reaction amplification of 12 highly polymorphic short-tandem-repeat markers spanning the entire length of chromosome 6. In the seventeen patients where relapse was confirmed to have a host origin, five patients had an epigenetic loss of the mismatched haplotype, allowing the escape of AML cells from alloreactive CD8+ and CD4+ T cells [23]. Importantly, despite a predicted susceptibility to NK cells due to the lost haplotype, NK cells were not able to prevent relapse in this setting, suggesting that more suppressive factors are driving innate resistance, or the numbers of functional NK cells are too low after relapse. These data suggest that knowing the HLA haplotype of the relapsed blast is critical to guide therapy decisions where a haploidentical donor lymphocyte infusion (DLI), composed almost entirely of T cells, would likely not be effective in treating or preventing AML relapse. Further investigation is needed to determine if therapy can change this immune strategy and augment future DLI efficacy. 

Strategies to overcome class II loss have been explored. In a phase I pilot study in patients with relapsed AML and MDS, IFN-γ followed by donor lymphocyte infusion (DLI) increased HLA-DR post-transplant and augmented GVL, and efficacy seen in the four patients (NCT04628338) [24]. Interferon-gamma also induces the expression of B7/BB-1 on monocytes, which is the ligand for the T-cell co-stimulatory CD28 pathway augmenting, T-cell activation and cytokine secretion [25]. Alternatively, interferon-gamma pathway activation has been associated with chemotherapy resistance, immune exhaustion, BCL2 inhibitors, venetoclax (VEN), and resistance in AML with high MHC class I and II expression, suggesting that this pathway has dual roles for immune activation and immune suppression [26]. A recent retrospective study completed by EBMT evaluated the outcomes of 173 patients with hematological malignancies (80 patients with AML) after haploidentical SCT- and PTCy-based graft versus host disease (GVHD) prophylaxis who received DLI for either relapse prevention or treatment. In the patients with hematological relapse, 2 year overall survival (OS) was only 22%, suggesting a need for better approaches to treat relapsed disease in this setting [27]. Adding interferon-gamma or sequencing of targeted therapy, such as VEN-based treatment, may rescue class II expression on blasts and augment DLI efficacy through T-cell-mediated interactions; however, further exploration is needed. Together, these studies suggest that HLA loss is a key driver of relapse but may be reversible with new therapeutic strategies, including the use of interferon-gamma.

2. Clonal escape/tumor heterogeneity/oncogenesis

Clonal escape and maturation shifts play a role in immune resistance. Genetic alterations can drive differentiation of the blast to a more mature phenotype with changes in cell surface and tumor antigen presentation. Single-cell sequencing (scRNA-seq) was performed on 35 bone marrow AML samples without enrichment to determine a broad examination of all cell subsets to determine the impact of the differentiation state of the blast, genetic alterations, and immune correlation [28]. Through the clustering of RNA sequencing data, the cell state of the myeloid blast was divided into six clusters, ranging from primitive to mature. Compared to healthy marrow samples, there were fewer total T cells and CTLs with a reduced CTL:T cell ratio in AML patients. In addition, the monocyte-like cells or mature AML cohort had an overexpression of TNF pathway genes (TRAIL/TNFSF10, TNFAIP2), IL-10 pathway genes (STAT1, HMOX1), and regulators of reactive oxygen species (TXNIP) [28]. These findings suggest that monocytic AML may be less sensitive to many immune-based therapies. Austin et al. analyzed the impact of oncogene NRAS G12D, seen in monocytic AML, and its impact on immune ligands. NRAS G12D was associated with increased MHC class II, CD28 ligands, CD80 and CD86 expression, and T-cell proliferation, while the expression of Myc was associated with less MHC class I and II expression [21]. These data suggest that T cells may be more sensitive to NRAS G12D mutant AML due to high MHC expression, but Myc-expressed tumors may be more sensitive to NK cells. In the clinic, resistance to VEN has been associated with monocytic maturation, RAS signaling pathway activation, TP53 mutations, and the downregulation of BCL2 [29]. This phenotype of VEN resistance also correlates with a distinct immune profile, likely mediated by interferon-gamma pathway activation and high MHC class I and II expression [26]. Based on prior data presented above, one would expect a similar immune resistance. Mature monocytes express less BCL2 than stem cells but also overexpress the checkpoint molecule LILRB4, which decreases T and NK cell function [30]. Conversely, immature stem cells have a high BCL2 expression but have a low NKG2DL expression, which leads to NK cell resistance. Studies have shown that this resistant phenotype can be rescued by PARP1 inhibition, which induces NKG2DL on leukemia stem cells [31]. These studies highlight a need for more personalized approaches to cellular therapy, accounting for the blast maturation state, genetic profile, and expected immune ligand pairs to select the correct immune therapy.

3. Immune Suppression (T and NK cells, MDSCs, dendritic cells)/Checkpoint

The immunosuppressive cancer stem cell niche plays a major role in dictating immune recognition and driving chemotherapy resistance. The bone marrow niche encompasses includes many cell types (stromal cells, blasts, dendritic cells (DC), myeloid-derived suppressor cells (MDSCs), NK cells, and T cells) that are capable of crosstalk between myeloid stem cells [32]. Multiple studies have shown that AML results in alterations of cytokines that are important for immune survival and lytic function [26,33,34]. These studies have revealed immunosuppressive DCs and macrophage subsets, exhausted and dysfunctional T/NK subpopulations, and suppressive T cells [35]. Specifically, checkpoint molecules are highly expressed in AML patients with functional consequences. T cell immunoglobulin and mucin domain 3 (TIM3) is an inhibitory checkpoint protein expressed on lymphocytes. TIM3 binds Galectin 9 (Gal9) to suppress immune function and prevent effector T cell function. Higher levels of TIM3 were seen in flow analysis of AML patients undergoing induction therapy and its presence predicted poor response or treatment failure [36]. LILRB4 is an inhibitory receptor expressed on mature monocytes that can inhibit T cell and NK cell function against leukemic blasts [37]. Antibodies are currently in trial to block LILRB4 to augment the immune killing of monocytes. IO-202 is an antibody for LILRB4 currently being studied in a clinical trial for patients with CMML or monocytic AML with or without the combination of a hypomethylating agent and VEN (NCT04372433) [30]. CD47 is commonly found on myeloid blasts and is an inhibitory receptor signaling “do not eat me” molecule. It blocks phagocytosis by binding to signal regulatory protein α (SIRPα). CD47 has been associated with an unfavorable prognosis, like the other checkpoint molecules described, and there are several clinical trials ongoing blocking CD47 [36,38,39,40,41,42]. In addition to antibodies to block checkpoints, many of these molecules are also being explored as antigens for CART or NK therapy. 

### 1.2. T Cell Dysfunction in AML

T cells are a crucial part of the adaptive immune system and a potent effector cell in treating leukemia. During development in the thymus, T cells become functional through T-cell receptor (TCR) gene rearrangement and selection through peptide MHC complex binding on antigen-presenting cells (APCs). In general, T cells can develop into two major groups: CD4+ T cells, which include regulatory T cells (Tregs), and CD8^+^ T cells. Tregs modulate the immune response through the generation of immunosuppressive cytokines that downregulate the activity of conventional T cells and subsequently facilitate tumor escape [43,44]. CD8+ T cells, on the other hand, can differentiate into cytolytic- and cytokine-producing effector cells [45]. These cytolytic effectors, also called cytotoxic T lymphocytes (CTLs), are capable of initiating tumor cell killing via the release of granzyme B and perforin. Though they might differ in their effector functions, both CD4+ and CD8+ T cells are capable of recognizing tumor-specific antigens via MHC class II or class I molecules, respectively, to trigger an immune response against tumors [46]. The interplay between CD4+ and CD8+ T cells represents an important mechanism of endogenous cancer surveillance. 

T-cell exhaustion and dysfunction in AML have been well described [47,48]. Tregs are enriched in the bone marrow niche, correlating with the secretion of immunoinhibitory factors, such as IL-10, IL-35, transforming growth factor-beta (TGF-β), and indoleamine 2,3-dioxygenase 1 (IDO1) from AML blasts [49,50]. The relative proportion of Tregs in AML is increased in AML patients, and this correlates with a worse prognosis in AML. Tregs also secrete immunosuppressive cytokine IL-10 [51,52]. Xie et al. profiled over 122 AML patient samples from RNA sequencing data obtained from The Cancer Genome Atlas (TCGA) and the Gene Expression Omnibus (GEO) database to try to obtain an algorithm for predicting response to immune therapy. Analysis of the genetic mutations led to two different groups with poor prognoses, with a higher expression of IL2RA, HAVCR2, and LAG3, while HAVCR2 expression was downregulated. The high-risk group also had more checkpoint expression on T cells, more relative Tregs, and received less benefit from immune effector therapy [47,48]. In addition, CD4+ and CD8+ T cell profiles in AML also show higher PD-1 expression and correlate with relapse disease post-transplant [43,44,53,54,55]. While a PD-1 blockade has been successfully used in solid tumors and classical Hodgkin lymphoma, its success rate in AML is diminished [56]. Hypomethylating agents (AZA or DAC) are frequently given to elderly patients not fit for intensive chemotherapy [57]. AZA treatment upregulates PD-1, suppressive Tregs, and IFNγ signaling [58,59]. Two clinical trials tried to combat the immune suppressive activity of AZA by adding the PD-1 inhibitor, nivolumab, or the PDL1 inhibitor, pembrolizumab, in relapsed/refractory AML patients [57]. The overall response rate was 35%, with 11 patients achieving complete remission in the nivolumab trial and 40% of patients having stable disease with pembrolizumab [60]. There was an increase in CTLA-4-positive CD8^+^ T cells over time, indicating that the upregulation of CTLA-4 could play a role in PD-1 therapy resistance and that combination therapy targeting both molecules might be necessary to achieve higher response rates [57]. Nivolumab was also studied in the post-allogeneic transplant setting in a randomized phase II study (REMAIN trial NCT02275533) of observation vs. nivolumab (3 mg/kg IV every 2 weeks for forty-six doses). Unfortunately, out of 79 patients enrolled, no differences were seen in progression-free survival (PFS) or OS with nivolumab maintenance over observation [61], suggesting that reversing T cell exhaustion through a PD-1 blockade alone is not enough to prevent relapse. When AZA was combined with DLI in a prospective single-arm multicenter phase II, the response rates were still round 30%, similar to AZA + PD1 or PDL1. The trial enrolled thirty patients as salvage therapy in relapsed AML or MDS after alloHSCT. It found that the combination was safe and effective at maintaining long-term remissions, with an overall response rate of 30%, including seven complete remissions (CRs, 23%) and two partial remissions (7%) [62].

Several studies have investigated the role of reversing the suppressed T cell phenotype using several small molecule inhibitors. A phase I clinical trial using Selinexor, a selective inhibitor of nuclear export compound that blocks exportin 1 (XPO1), in combination with Cytarabine and Mitoxantrone in 26 AML patients, found a higher frequency of PD-1+ CD4+ and CD8+ T cells in treatment-failure patients than in those achieving complete remission. Additionally, the percentage of Gal-9^+^CD34^−^ cells was significantly higher in total-failure patients, and an increased expression of Gal-9 was correlated with the expression of immune checkpoint molecules TIM-3 and Lag3 [36]. This is particularly interesting since a study conducted on TIM-3/Gal9 signaling showed that they form an autocrine loop that is critical for leukemic cell self-renewal and the development of AML [63]. 

The dysfunction of the immunological synapse between T cells and AML blasts can also contribute to immune resistance [64]. One study investigating the formation of this synapse found that AML blasts not only influence T cells via the upregulation of inhibitory ligands but also change gene expression profiles by subjecting blasts to an aberrant activation process. This study, therefore, showed that while T cells can recognize and interact with AML cells, they may fail to induce effector functions and facilitate target killing [65]. 

### 1.3. NK Cell Dysfunction in AML

NK cells are both innate and adaptive and have the ability to recognize and kill both virally infected and malignant transformed cells [66,67]. NK cells make up 10% of lymphocytes in the peripheral blood and are almost exclusively CD56 dim, CD16-positive mature NK cells. Several studies have shown the potential of NK cells in inducing lasting remissions in AML [68,69,70]. Unlike T cells or B cells, NK cells are not antigen-restricted and do not cause autoimmunity or GVHD, making them a safer and readily available “off the shelf” allogeneic product for patients [71,72]. NK cell function is controlled by germline-encoded receptors, which are either activating or inhibitory. The “balance” or “lack of balance” between the receptor/ligand pairings induces a positive or a negative signal determining NK cell action [73]. The killer-cell immunoglobulin-like receptors (KIRs) are the most recognized and studied NK cell receptors capable of recognizing classical HLA class I molecules (HLA-A, -B, and -C). KIRs can be inhibitory (iKIR) or activating (aKIR) and bind to specific MHC class I epitopes. KIR2DL1 (CD158a) and KIR2DL2/2DL3 (CD158b) recognize HLA-C epitopes, while KIR3DL1 (CD158e1) recognize HLA-A and HLA-B allotypes that express the Bw4 epitope [67]. When healthy cells are present, NK cell-mediated lysis does not occur due to the recognition of the “self” through HLA class I binding to the iKIR. However, in the setting of malignant or viral-infected cells, HLA is downregulated or missing, and the iKIR is not bound, activating NK-mediated killing [17]. Other surface receptors include leukocyte immunoglobulin-like receptors (LIRs) and the C-type lectin family (CD94 and NKG2s, including NKG2A, -B, -C, -D, -E, and -F), which recognize non-classical class I molecules (HLA-E, F, MICA/B). The NKG2D (natural killer group 2, member D) lectin-like receptor is a primary mediator of enhancing NK cell function [74]. NKG2D is expressed abundantly on all NK cells and binds to NKG2DL on tumor cells to induce NK cell lysis [75]. NKG2A is a lectin-like receptor expressed on immature NK cells and has been shown to mediate primarily inhibitory effects upon binding its target, human leukocyte antigen-E (HLA-E) [76]. Activating receptors include heterodimer CD94/NKG2C, NKG2D, DNAM-1, CD16, and natural cytotoxicity receptors (NCRs), including NKp30, NKp44, and NKp46 [73,77]. CD16 or FcγRIIIA, expressed on mature NK cells, is a critical mediator of antibody-dependent cellular cytotoxicity (ADCC) by binding to the Fc portion of antibodies and triggering direct cell lysis [67]. NK cells also have adaptive or memory properties (defined as CD57+NKG2C+) and are generated from prior CMV exposure [78]. These cells have been shown to have enhanced cytolytic activity against tumors, and expansion of these cells post-alloHSCT has shown protection from relapse [79]. Cooper et al. described these cells in 2009, showing that the pre-activation of peripheral blood-isolated NK cells exposed to a cytokine cocktail of IL-12, IL-15, and IL-18 induces differentiation toward a memory-like NK cell with enhanced effector function and persistence [80].

**Figure 2:**
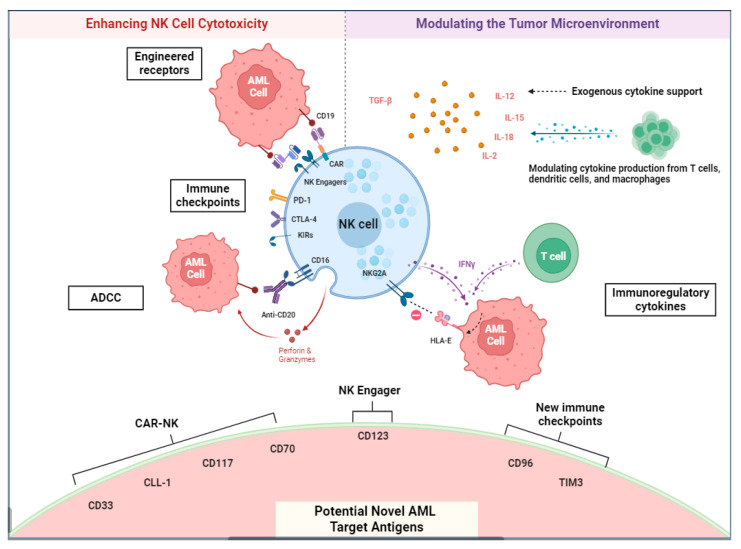
NK cell targets in AML. Strategies aimed at enhancing NK cell cytotoxicity include the development of engineered receptors, targeting immune checkpoint molecules, and enhancing antibody-dependent cytotoxicity (ADCC). The tumor microenvironment of AML uniquely constitutes activating and suppressive cytokines, and the modulation of these cytokine signaling pathways represents a potentially novel strategy for enhancing NK cell activity. Lastly, a variety of lineage-specific surface antigens are being explored as potential targets for NK cell-based therapies, including CAR-NK therapy, NK cell engagers, and immune checkpoint blockades. This figure was created with Biorender.com.

Alloreactive NK cells are the most potent and active against tumors. In the haploidentical transplant setting, the donor KIRs and the recipient HLA class I are not HLA matched, making the NK cell more activated against the discrepant host leukemia haplotype. Alloreactive NK cells have been shown to drive long-term remissions in the setting of haploidentical transplant [81]. In recent years, ex vivo expansion protocols have enabled single-donor allogeneic NK cells with the capability of repetitive dosing [82,83]. As such, efforts are ongoing to harness the cytotoxic capabilities of NK cells as a safer form of immunotherapy for several hematological malignancies, including AML. Despite many advancements in the field, the immunosuppressive tumor biology and microenvironment of AML continue to represent a challenging barrier in optimizing NK cellular therapy (Figure 2). 

The complex biology of AML encompasses a myriad of pathways that can facilitate alterations in NK cell function. Stringaris et al. evaluated NK cell phenotype and function in 32 patients with newly diagnosed AML, including 12 patients achieving complete remission [84]. They found that NK cells from AML patients exhibited a downregulation of activating receptor NKp46 and an upregulation of inhibitory NKG2A. These changes translated into impaired degranulation against autologous blasts and K562 targets, with significantly reduced CD107a degranulation, TNF-α, and IFN-γ production [84]. NK cell expression of NKG2A correlated with failure to achieve remission and was partially revered with treatment. In another study of 21 untreated AML patients, Sandoval-Borrego et al. isolated peripheral blood NK cells and performed immunophenotyping based on French–American–British (FAB) morphology [85]. The authors found that the differentiation state, based on FAB, impacted NK cell receptor expression. For example, patients with AML M3 (acute promyelocytic leukemia) showed a decreased expression of the activating NKG2D receptor, while all AML patients displayed an overexpression of the inhibitory receptors CD158b and NKG2A compared to healthy controls [85]. Other studies have shown that the downregulation of NCRs represents a mechanism of defective NK cell activity in AML [86,87,88]. Costello et al. examined NK cell function in a cohort of 18 patients with AML by analyzing the expression pattern of various NCRs [88]. They found a low expression of the activating receptors NKp46, NKp30, and NKp44 in 16 out of 18 patients with AML. Functional studies revealed that AML-NK cells characterized by the NCRbright phenotype recognized allogeneic blasts with strong cytolytic activity; however, the NCRdull or low-expressing NCRs failed to kill autologous leukemic cells. Autologous killing was not rescued in the presence of anti-HLA class I mAbs; however, it was rescued when anti-NKp46 or anti-NKp44 mAb was in the NCRbright NK cell population [88]. 

Along with the direct alteration of host NK cell phenotype summarized above, NK cell evasion in AML may also be mediated by tumor-intrinsic factors, transcription factors, and epigenetic changes that block immune cell maturation. Several studies have shown that immature NK cell profiles are associated with poorer overall survival in AML patients [89,90]. Chretien et al. performed flow as well as mass cytometry to reveal that the NK hypomaturation profile was associated with a reduced frequency of memory-like NK cells, which are thought to be the most active cells against leukemia [91]. Scoville et al. further described the NK cell differentiation block in AML patients, with a mechanism highlighting a direct role of the transcription factor, the aryl hydrocarbon receptor (AHR), secreted from AML blasts (likely via exosomes) that was reversible with AHR inhibition [92]. Similarly, CD200 is an immunosuppressive glycoprotein known to be upregulated in AML and is associated with less NK cell maturation and poor clinical outcomes [79]. Coles et al. showed that CD200 overexpression in AML led to compromised NK cell-mediated tumor responses. The authors separated AML patients into two groups, CD200hi and CD200lo, with CD200hi patients having a 50% reduction in the frequency of activated, mature NK cells (CD56dimCD16+) with impaired function compared with CD200lo patients [93]. The mechanism associated with CD200 expression on AML blasts and less NK cell maturation or activation was not explored; however, the authors found that a blockade of CD200 was able to rescue defective NK cell cytotoxicity in CD200+ AML [93]. 

The immune checkpoint profile of NK cells also plays a critical role in AML immune resistance and was recently studied by Liu et al. [94]. They found that in newly diagnosed AML patients, NK cells exhibited an increased expression of PD-1, TGIT, and TIM-3 compared to healthy donors, like T cells [94]. Further analysis showed that TGIT^+^ NK cells were impaired functionally, with lower cytotoxicity against leukemic blasts and decreased cytokine production, and a blockade of TIGIT could reverse this dysfunctional phenotype [94]. Several other immune checkpoints have been shown to suppress NK cell activity, such as CTLA-4, B7-H3, LAG-3, and CD96, and are currently being studied as potential therapeutic targets for restoring NK cell cytotoxicity [95]. 

The AML TME is also known to suppress NK cell activity through a variety of mechanisms [35,71]. Nguyen et al. showed that IFN-γ derived from immature NK cells upregulated the expression of HLA-E in AML patients after haploidentical HCT and that the subsequent defective NK cell responses could be restored by blocking NKG2A [76]. Another recent study by Wang et al. identified that the TME of monocytic AML uniquely expressed increased levels of IFN-γ produced by NK cells and T cells, and IFN-γ signaling scores correlated with VEN resistance and immune evasion [26]. The IFN-γ axis thus represents a potential target within the AML TME for modulating immune responses. Tumor growth factor β (TGF-β) is another cytokine abundant in the TME that promotes NK cell inhibition through altering activating receptors and IFN-γ production [33]. Studies utilizing genetically modified iPSC-derived NK cells with disrupted TGF-β transcription have demonstrated enhanced NK cell proliferation and scalable manufacturing [96].

4. Tumor Microenvironment

The bone marrow niche or tumor microenvironment plays a major role in the development and progression of AML [12]. The TME encompasses a complicated network of cell types, including stromal cells, immune cells (B, T, NK cells, antigen-presenting cells (APCs), and tumor-associated macrophages (TAMs)). The TME facilitates signaling among blasts and neighboring cells to alter homeostasis through the secretion of inhibitory factors, such as IL-10, growth factor-beta (TGF-β), and IL-35, which are all responsible for driving T cells toward a Treg phenotype and limiting T cell proliferation. TGF-β is a cytokine that has important roles in leukemic proliferation, with lower levels found in AML patients, leading to enhanced leukemogenesis [33]. IL-35 is a cytokine that belongs to the IL-12 family and is secreted by several types of cells, including Tregs, monocytes, dendritic cells, and macrophages. IL-35 is associated with tumor progression in addition to impaired T cell recognition and function [97]. Myeloid-derived suppressor cells (MDSCs) are myeloid cells that originate from bone marrow stem cells and expand under stress conditions such as infection, autoimmunity, chronic inflammation, or malignancy [98]. In studies, the functional role of MDSCs can be tested by depleting antibodies against two main proteins expressed in MDSC, including GR1 or lymphocyte antigen 6 complex locus G6D (Ly6G) [98]. Interestingly, MDSCs in melanoma have shown that they can acquire ten–eleven translocation 2 (TET2) mutations through signaling of interleukin-1 receptor (IL-1R), leading to immune function defects [99]. MDSCs also drive immune evasion by transforming normal macrophages into tumor-associated macrophages (TAMs), which contribute to hypofunctional immune responses. Healthy activated macrophages can be either the M1 or M2 type. M1 macrophages secrete IL-1, IL-6, IL-12, tumor necrosis factor-α (TNF-α), CXCL9, CXCL10, nitric oxide, and reactive oxygen species consistent with a pro-inflammatory signature. The M2 macrophages secrete IL-10, transforming growth factor-β (TGF-β), CCL17, CCL22, arginase, and mannose (CD206), which are thought to be more tumor promoting [100]. TAMs resemble the M2 phenotype, and higher levels have been correlated with worse prognosis in AML patients [100]. Indolamine-2, 3-dioxygenase (IDO) is a catabolizing enzyme of tryptophan and is involved in immune regulation. It is expressed by APCs, including macrophages and dendritic cells, but can also be secreted directly from stem cells. IDO has been shown to block T-cell activation. IDO metabolites, including kynurenine, are also directly toxic to both T and NK cells. Several IDO inhibitors exist; however, clinical trials have not shown significant activity as a single agent but remain to be considered as an addition to cellular therapy [101]. Arginase II is a manganese-containing enzyme, secreted by AML blasts, which differentiates monocytes toward an immunosuppressive M2-like phenotype and inhibits the proliferation of T cells and CD34+ hematopoietic stem cells. The inhibition of arginine metabolism through small molecule inhibitors restored stem cell proliferation but was thought to be too toxic to move into clinical trials [102]. Overall, reversing the TME suppressive phenotype through the depletion of Tregs, MDSCs, or cytokines may become an important mechanism to augment current treatment strategies soon.

The evolving knowledge of the genomic heterogeneity of AML and the implications for the TME has led to advances in T cell- and NK cell-based therapy, and in the following sections, we review current immune therapy and end with future directions. 

## 2. Current Immunotherapies in AML 

### 2.1. T-Cell-Based Immunotherapy

#### Chimeric Antigen Receptor T Cells 

Chimeric antigen receptor T cells (CAR-T) are genetically modified T cells engineered to express tumor antigens and have been successfully employed in a variety of solid and lymphoid malignancies. AML has remained a challenging disease to target with CAR-T therapy due to its molecular heterogeneity and commonly shared surface antigens with normal hematopoietic stem cells [103,104]. 

There are currently eight ongoing CAR-T clinical trials for AML patients (per clinicaltrials.gov) (Table 1). Multiple early-phase clinical trials have investigated CD33, CD123, and CD38 as potential CAR-T targets in relapsed and refractory AML, with varying response rates and degrees of toxicity [103,105]. A single-center phase I trial was performed using autologous T cells, modified to express a CD33-targeted CAR with 4-1BB and CD3 co-stimulatory domains co-expressed with a truncated human epidermal growth factor receptor for relapsed/refractory AML patients. In total, ten patients were enrolled, but only four of the eight patients were able to have CAR-T cells that met the release criteria for infusion [106]. Due to rapid disease progression in one patient, only three patients were infused, and none of the infused patients responded. In addition, the therapy was associated with known toxicity, including two patients who developed cytokine release syndrome (CRS) and one patient who developed immune effector cell-associated neurotoxicity syndrome (ICANS) [106]. The authors concluded that lentiviral transduction was difficult in AML patients and was only feasible in patients with a higher lymphocyte count and a lower blast count. CD123-directed CAR-T therapy was investigated in another phase one trial in refractory AML patients after allogeneic stem cell transplant. The study included six patients, with one patient in the first dose level achieving a morphologic leukemia-free state (MLFS) lasting 2 months and another patient achieving CR in DL2 who received another transplant at day 70 post-CAR-T [107]. Another phase I trial evaluated CD38-CAR-T in relapsed AML patients post-alloHSCT. The results showed CR or CR with incomplete count recovery (CRi) in four out of the six patients (67%), with a median CR or CRi time of 191 (range 117–261) days. Of note, all six enrolled patients had a high expression of CD38 with a median of 95% (92–99%) positivity [108]. The human C-type lectin-like molecule-1 (CLL-1) is another promising target that is expressed on AML blasts but not on healthy hematopoietic cells [109]. In a phase I clinical trial utilizing CLL-1 CAR-T in relapsed/refractory AML, seven out of the ten (70%) patients achieved CR or CRi. Despite the initial response rates, the treatment was toxic, with most patients experiencing high-grade CRS and two patients dying of severe infection [110]. CD7 is a transmembrane glycoprotein and a member of the immunoglobulin supergene family expressed on T and NK cells. It is expressed in approximately 30% of adult AML cases and is associated with a worse overall survival [111]. Although it appears to be a promising target, CD7-directed CAR-T therapies have impaired the expansion of transduced T cells due to residual CD7 expression, leading to fratricide [112]. Genomic disruption of CD7 on the T cells prior to CAR transduction allows for the generation of CD7 CAR-T cells without extensive self-antigen-driven fratricide and may be a viable option for overcoming this major limitation [113]. Other AML target molecules undergoing investigation as potential CAR-T-directed antigens include FLT3, NKG2D, and CD70 [105,114]. Despite small sample sizes, these early-phase studies show the promising potential of CAR-T therapy in AML. Strategies to reduce off-tumor effects, expedite manufacturing, and address or reverse the suppressive effects of the AML microenvironment are being explored to expand the landscape of CAR-T therapy for AML. 

### 2.2. Antibody-Based Therapies

Although several monoclonal antibodies have been developed for AML, few are being used clinically outside of a clinical trial. Mylotarg (Gemtuzumab ozogamicin; GO) is a humanized anti-CD33 monoclonal antibody drug conjugated with calicheamicin derivative and is the only FDA-approved antibody for use in CD33-positive AML [115]. It is currently approved in combination with induction chemotherapy (primarily in favorable risk AML) or first relapse in patients 60 years of age or older who were not candidates for cytotoxic chemotherapy. The addition of GO to induction 7+3 chemotherapy for newly diagnosed AML was associated with improved event-free survival (EFS) for cytogenetically favorable-risk AML. Major toxicity associated with GO is veno-occulsive disease (VOD), especially in patients who received GO before or after alloHSCT [116]. Other monoclonal antibodies under investigation include Talacotuzumab (CSL362) and tagraxofusp (SL-401), which target CD123, another commonly expressed antigen on AML blasts and not normal hematopoietic cells [117]. CD70 is a glycoprotein expressed on myeloid blasts [118]. A phase I/II clinical trial was completed using cusatuzumab (anti-CD70 monoclonal antibody) combined with azacitidine in patients with newly diagnosed AML who were ineligible for intensive chemotherapy. There were 38 patients in phases I and II, with 14/38 achieving complete remission with an acceptable safety profile [119]. 

Bispecific T-cell engagers (BiTEs) are recombinant antibodies that recruit T cells through CD3 engagement combined with a tumor antigen-targeting antibody [9]. Several bispecific antibodies targeting AML antigens are being investigated in ongoing early-phase trials for relapsed/refractory AML, including agents directed against CD33, CD123, CLL-1, and FLT3 [9,120]. The preliminary results indicate that BiTEs have an acceptable safety profile and may represent a promising therapy for relapsed/refractory AML [120]. CRS is a common adverse effect from these early-phase studies and, therefore, these treatments need to be administered in tertiary care centers with appropriate supportive care [120,121]. The dual-affinity retargeting antibodies, DARTs, are antibodies that have a longer half-life than BiTEs due to the manufacturing of two Fv fragments with increased stability [122]. A multicenter, open-label, phase I/II study of Flotetuzumab, a CD123xCD3 DART administered by continuous IV infusion, enrolled 88 adults with relapsed/refractory AML and showed an overall response rate (CR/CRh/CR with incomplete hematological recovery) of 30% and a median overall survival of 10.2 months [123]. Notably, this treatment was associated with both CRS and infusion-related reactions, of which most were minimal (grade 1–2). These initial results suggest great promise but also significant and predictable toxicities that require quick identification and the prompt use of specialized treatments such as tocilizumab, steroids, and aggressive supportive care. 

### 2.3. NK Cell-Based Immunotherapy

#### NK Chimeric Antigen Receptors and Other Methods of Adoptive NK Cell Transfer

The therapeutic potential of NK cells in AML was first recognized through their ability to mediate graft versus leukemia (GVL) effects in the context of alloHSCT [80]. Ruggeri et al. showed that donor–recipient KIR/HLA incompatibility in alloHSCT could be an effective method of harnessing NK cell alloreactivity [80]. In their study, 20 patients with AML received transplants from NK cell alloreactive donors, resulting in a 0% relapse rate at 5 years and no graft rejection or GVHD [80]. KIR and HLA mismatching between donor and recipient has since been recognized as an important predictor of OS, with benefit driven by fewer relapses seen in the KIR/HLA mismatch versus the KIR/HLA match in haploidentical transplants [124]. Miller et al. subsequently evaluated the safety and efficacy of adoptively infusing donor NK cells in a non-transplantation setting [125]. In this study, the authors showed that patients with AML who received a high-intensity lymphodepleting regimen of cyclophosphamide and fludarabine followed by an infusion of haploidentical, related-donor NK cells with subcutaneous IL-2 resulted in a marked endogenous production of IL-15 and donor NK cell expansion [125]. This substantial in vivo proliferation of NK cells correlated with anti-tumor effects, with complete hematologic remission observed in five of the nineteen patients with poor-prognosis AML [125]. More recently, cytokines have been used ex vivo to expand NK cells and activate them with improved function. A first-in-human phase I clinical trial in adult AML patients showed that the adoptive transfer of HLA haploidentical, IL-12, IL-15, and IL-18 pre-activated NK cells was safe and feasible with peak persistence between day 7 and day 14. Of the nine evaluable patients, four achieved CR/Cri, and one achieved MLFS, with an encouraging overall response rate of 55% [126]. In general, these cells were well tolerated but did not have sustained remission, which was likely related to a lack of NK cell persistence over time. Further efforts are underway to determine the correct dose, frequency, and cytokine support needed to improve cell proliferation and in vivo survival. 

CAR-T cells and NK cells can be expanded and engineered to express certain antigen receptors that enhance recognition and killing of tumor targets [127]. CAR-NK cells are an emerging cellular therapy product with distinct advantages compared to CAR-T cells [10,128]. Most notably, because NK cells do not rely on MHC-restricted antigen recognition, they avoid the potential therapy-limiting morbidity of GVHD [129]. Indeed, early-phase CAR-NK studies have been associated with low rates of GVHD, CRS, and immune effector cell-associated neurotoxicity syndrome (ICANS) [130]. Current studies utilizing CAR-NK therapy for AML involve targeting AML-specific antigens, such as CD33 and CD123 [131]. Persistence of CAR-NK cells remains a major limiting therapeutic hurdle, with studies showing that CD19 CAR-NK cells for lymphoma typically persist in the recipient for less than 12 months [132]. Of note, CAR-NK used in Liu et al.’s study was given fresh without cryopreservation, while a phase II study using the same CAR-NK CD19 cryopreserved and thawed at the bedside is ongoing. Further studies to enhance CAR-NK efficacy include constructing CARs against novel targets, such as fms-like tyrosine kinase 3 (FLT3), developing NK-specific co-stimulatory domains, implementing immunomodulatory cytokines to recruit other effector cells, utilizing cytokines to improve in vivo expansion, and employing dual CAR-NK targeting [128,129]. 

Umbilical cord blood is a viable source of NK cells and is readily available [133,134]. Although umbilical cord NK cells exhibit an excellent safety profile, their immature phenotype has led to inconsistent efficacy and reduced cytotoxicity [135]. Immature NK cells from cord blood have been shown to express lower functional levels of perforin and granzyme, along with increased expression of the inhibitory receptor NKG2A [136]. These limitations of cord blood-derived NK cells have spurred research into more reliable methods of adoptive NK cell therapy. For example, induced pluripotent stem cells (iPSCs) are isolated from umbilical cord blood and expanded and modified to express transcription factors critical for maintaining pluripotent stemness [137]. iPSC-derived NK cells exhibit high levels of proliferation and anti-tumor cytotoxicity [137]. A recent study by Chiu et al. showed that iPSC-derived NK cells engineered to express the activating memory NK cell receptor, NKG2C, demonstrated enhanced cytotoxicity against AML blasts [138]. Lastly, iPSCs have also been engineered to express a non-cleavable CD16 receptor, which would prevent “CD16 shedding”, a phenomenon seen after CD16 is activated, to enhance repetitive ADCC. Preclinical investigation shows that this iPSC line administered with anti-CD20 mAB-mediated potent activity and improved long-term survival in a mouse lymphoma xenograft model [139]. 

### 2.4. NK Cell Engagers

Similar to T-cell engagers, NK cell engagers bind tumor antigens and NK cell receptors to facilitate recruitment and tumor-directed cytotoxicity [140]. Bispecific killer engagers (BiKEs) are constructed with two single-chain variable fragments that bind to an activating NK cell receptor and a tumor-specific antigen [140]. Trispecific killer engagers (TRiKEs) are BiKEs that are formulated with an additional variable fragment to augment cytotoxicity [141]. Compared to CAR-NK cells, NK cell engagers are an “off the shelf” product without additional manufacturing needed. In a high-risk patient population, such as relapsed hyperproliferative AML, these therapies have an advantage of being readily available without the need for individual production [128]. Current early-phase trials are investigating NK cell engagers in AML utilizing the NK cell receptor CD16 and the AML antigen CD33 [142]. One major limitation was the in vivo expansion and persistence of NK cells. To improve persistence, TRiKEs have been developed with the addition of a novel human IL15 crosslinker with a single-chain scFv against CD16 and CD33 to create a stronger immunologic synapse between NK cells and CD33(+) myeloid targets for improved efficacy [141]. The TRiKEs had a lot of hope given their superior cytotoxicity to AML cell lines and enhanced NK cell proliferation in mouse models over BiKEs; however, they have not shown many clinical responses. Unfortunately, a phase I clinical trial testing this compound (GTB-3550) in R/R AML and high-risk MDS patients, administered by continuous infusion, provided no objective responses despite leading to NK cell proliferation in all patients [143]. This suggests that the TME may be playing a role in limiting NK cell response and effort to restore a healthy cytokine and immune profile and may improve responses. SAR’579 is a trifunctional NK cell engager targeting the CD123 antigen (expressed commonly on AML cells) and co-engaging with the NK cell-activating receptor (NKp46) and CD16a on NK cells. The clinical trial is currently enrolling patients with multiple CD123-expressing tumors, including R/R AML, MDS, and B-ALL (NCT05086315), and the initial results show encouraging efficacy, with two patients achieving a CR/CRi [144]. 

## 3. Conclusions

Our enhanced knowledge of the mechanisms mediating immune evasion in AML has led to major transformations in the therapeutic landscape of cellular therapy for AML. Inherent differences in genetic and epigenetic regulation of AML and TME dictate vulnerability to immune-based recognition. This budding experience and knowledge have also revealed the limitations inherent to T cells and NK cells, emphasizing the need for improved cytotoxic immune cell-specific platforms. Since the initial studies describing the graft versus leukemia benefits of allogeneic NK cells in KIR-mismatched transplantation and the benefit of alloreactive donor T cells, multiple studies have expanded on the adoptive potential of immune-based therapy. The strategic use of immunotherapies for subsets of AML patients will become more commonplace. Promising advances have been made utilizing cytokine combinations and genetically engineered CARs to leverage T cell and NK cell cytotoxicity as an effective cellular therapy platform. Despite these discoveries, several limitations and questions remain unanswered. Increasing immune cell persistence, finding the optimal dosing strategy, determining the correct cytokine support, and optimizing the TME for cellular response are all unmet questions in the field. Future studies should seek to further understand the nuances of the immune microenvironment and its cytokine profiles to allow for the individualization of therapy and ultimately improve patient outcomes. 

## Figures and Tables

**Table 1:** CAR-T clinical trials for AML.

CART Target Antigen	Trial Name	Number
CD38	CART-38 in Adult AML and MM Patients	NCT0544280
CLL1	Anti-CLL1 CART-cell Therapy in CLL1 Positive Relapsed/Refractory AML	NCT04884984 NCT04923919
CD19	CART-19 T Cell in CD19 Positive in R/R AML	NCT03896854
CD33	CD33KO-HSPC-Infusion Followed by CART-33 Infusion(s) for R/R AML	NCT05945849
FLT3	Anti-FLT3 CAR T-cell Therapy in FLT3 Positive R/R AML	NCT05023707
CD7	Dose-Escalation and Dose-Expansion Study to Evaluate the Safety and Tolerability of Anti-CD7 Allogeneic CAR T-Cell (WU-CART-007) in CD7+ Hematological Malignancies	NCT05377827
CD33, CD38 CD56, CD117, CD123, CD34, and Muc1	CAR-T Cells Combined with Peptide Specific WT1 Dendritic Cell in Relapsed/Refractory Leukemia/MDS	NCT03291444
